# Nanoparticles-Based Delivery Systems for Salicylic Acid as Plant Growth Stimulator and Stress Alleviation

**DOI:** 10.3390/plants12081637

**Published:** 2023-04-13

**Authors:** Vladimir Polyakov, Tatiana Bauer, Vera Butova, Tatiana Minkina, Vishnu D. Rajput

**Affiliations:** 1 The Smart Materials Research Institute, Southern Federal University, 344090 Rostov-on-Don, Russia; 2 Academy of Biology and Biotechnology, Southern Federal University, 344090 Rostov-on-Don, Russia; 3 Institute of General and Inorganic Chemistry, Bulgarian Academy of Sciences, 1113 Sofia, Bulgaria

**Keywords:** salicylic acid, phytohormone, nanoparticles, delivery systems, plant defense, systemic acquired resistance

## Abstract

The population growth tendency leads to an increase in demand for food products, and in particular, products obtained from the processing of plants. However, there are issues of biotic and abiotic stresses that can significantly reduce crop yields and escalate the food crisis. Therefore, in recent years, the development of new methods of plant protection became an important task. One of the most promising ways to protect plants is to treat them with various phytohormones. Salicylic acid (SA) is one of the regulators of systemic acquired resistance (SAR) signaling pathways. These mechanisms are able to protect plants from biotic and abiotic stresses by increasing the expression of genes that encode antioxidant enzymes. However, salicylic acid in high doses can act as an antagonist and have the negative rebound effect of inhibition of plant growth and development. To maintain optimal SA concentrations in the long term, it is necessary to develop systems for the delivery and slow release of SA in plants. The purpose of this review is to summarize and study methods of delivery and controlled release of SA in a plant. Various carriers-based nanoparticles (NPs) synthesized from both organic and inorganic compounds, their chemical structure, impacts on plants, advantages, and disadvantages are comprehensively discussed. The mechanisms of controlled release of SA and the effects of the use of the considered composites on the growth and development of plants are also described. The present review will be helpful to design or fabricate NPs and NPs-based delivery systems for salicylic acid-controlled release and better understating of the mechanism of SA-NPs interaction to alleviate stress on plants.

## 1. Introduction

Meal shortages were predicted to be a crucial problem in 2050 as a response to population growth and, therefore, an increase in demand for foodstuffs [1]. Plants are one of the primary sources of food consumed by humans both in raw and processed form. In addition, plants are also a source of a large number of medicinal compounds belonging to various classes: alkaloids, terpenoids, flavonoids, essential oils, carbohydrates, and vitamins. According to scientists, to prevent the demand for foodstuffs problem, it is necessary to increase agricultural productivity by at least 70% [2]. However, about 40% of food crops are affected by pests and diseases exacerbated by climate change [3]. One of the main causes of yield losses is abiotic and biotic stress [4]. Abiotic stress is a reaction of plants to non-optimal external conditions, such as drought, abrupt changes in temperature, deficiency of mineral compounds, etc. Such conditions can lead to the suppression of growth, development, and also the genetic potential of plants due to metabolic disorders, changes in the molecular and biochemical nature of plants [5]. Biotic stress factors include the infection of plants with various pathogens: bacteria, viruses, and fungi. They also have a significant effect on the viability of plants. Therefore, finding effective ways to accelerate plant growth and their protection, including under biotic and abiotic stress, is one of the scientific development prospects.

**Figure 1:**
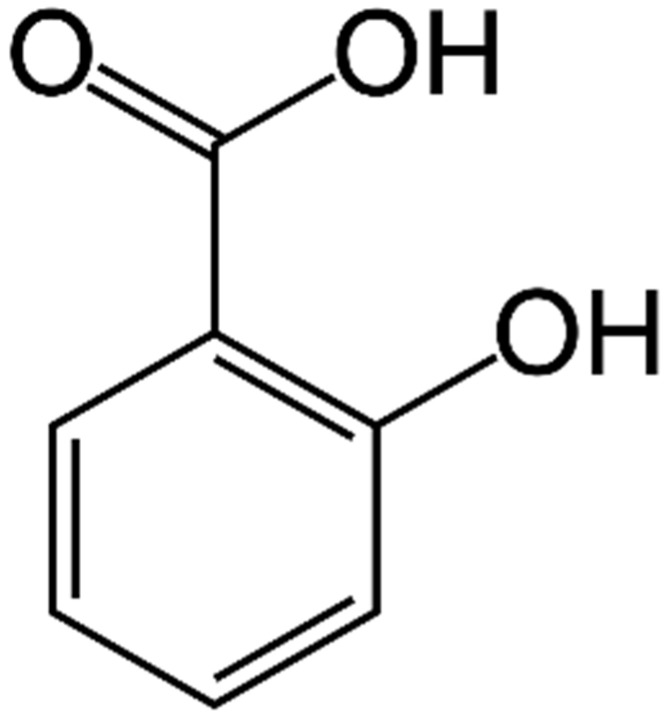
Salicylic acid structure.

In agriculture, various methods of accelerating plant growth are used, such as a genetic increase in plant resistance to pests and climatic conditions [6], the use of nanobionics [7], and applying fertilizers to the soil [8,9]. Phytohormones are increasingly used in cutting-edge technologies for stimulating plant growth and crop production. Small doses of these natural and synthetic organic compounds actively affect the metabolism of plants, causing stimulation or suppression of their growth and morphogenesis [10,11,12,13]. Such compounds also allow for reducing the amount of toxic chemicals used to protect plants from infections [14]. An essential criterion for the large-scale implementation of this technology is the availability of the phytohormone and the low cost of its production. One such substance is salicylic acid (SA). SA is a phytohormone of the phenol class (Figure 1).

SA plays an essential role in signaling pathways in systemic acquired resistance (SAR). SAR is a mechanism by which plant protection is pre-treated. As a result, plant resistance to various pathogens increases. SA helps plants protect themselves from biotic and abiotic stresses by increasing the expression of genes that code for antioxidant enzymes, which is just part of the mechanism of action of SA. SA is perceived by NPR1, a transcriptional activator [15]. In tomatoes, SA was found to induce seven stress-sensitive genes [16]: ethylene-responsive transcription factor 3 (RAP) [17], xyloglucan endotransglucosylase 2 (XET-2) [18], catalytic hydrolase-2 (ACS-2) [19], proteinase inhibitor II (PINII) [20], phenylalanine ammonia-lyase 5 (PAL5) [21], lipoxygenase D (LOXD) [22], and pathogenesis-related protein 1 (PR-1) [23]. However, the authors of this study did not test for NPR1 involvement. These genes are also closely related to drought tolerance-associated genes. Thus, for example, the genes of the WRKY family are among the most significant genes encoding transcription factors and are important in the abiotic stress tolerance, plant growth, and development [24,25]. They may play a role in responding to hormonal signals by controlling the expression of target genes [26,27]. Mitogen-activated protein kinases (MAPKs) regulate cellular signaling and, therefore, the ability to adapt to stress [28], late embryogenesis-abundant proteins (LEA) enhance the protection of cells from drought damage [29,30,31], and MYC transcription factors regulate crosstalk between jasmonic acid and other phytohormone signaling pathways [32].

**Figure 2:**
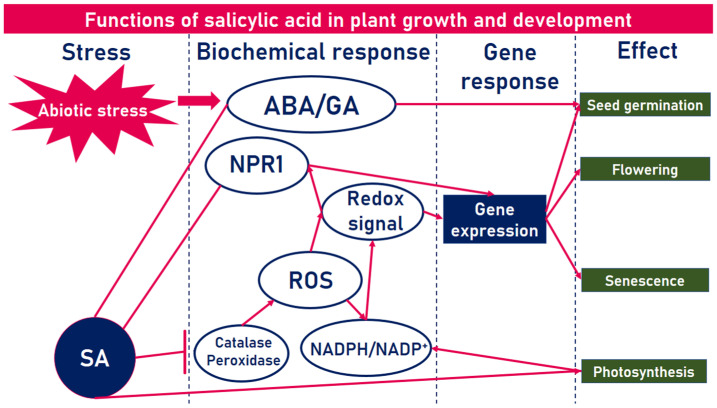
Functions of salicylic acid in plant growth and development. ROS–reactive oxygen species, ABA–abscisic acid, GA–gibberellins, NPR1–gene nonexpressor of PR-1, and NADPH/NADP+–a protonated and deprotonated form of nicotinamide adenine dinucleotide phosphate, respectively. Adapted from [48].

SA is also a natural trigger for heat production in thermogenic and non-thermogenic plants, promotes CO_2_ assimilation [33], regulates homeostasis [34,35,36,37], reactive oxygen species (ROS) levels by inhibiting catalase and peroxidase activity [38,39], stomatal conductance [40,41], photosynthesis [33,42], seed germination rate [43,44], an increase in the content of carotenoids and chlorophyll [33,45,46], and is involved in the accumulation of proline [47]. Thus, the availability of SA is necessary to maintain plant metabolism (Figure 2).

In addition, SA is a large-scale product, which has a positive impact on plants with the possibility of its widespread use in agriculture. However, the use of exogenous SA in a wide range of concentrations leads to variability in growth and yield. Often, high concentrations of SA lead to the inhibition of plant growth and development. The antagonistic effects of SA probably cause this reaction in plants [44,45,46,49,50,51,52,53,54]. Therefore, the main issue with the effective use of SA is its controlled release. For a prolonged sustainable effect of SA on plants, it is necessary to create conditions for the delivery and slow release of the phytohormone.

## 2. Salicylic Acid Delivery Systems

Both organic and inorganic nanoparticles (NPs) are used as a carrier for delivering salicylic acid (Table 1).

### 2.1. Chitosan-Based Delivery Systems

Chitosan (CS) is most often used as a delivery matrix. This is a biocompatible heteropolysaccharide built from 1,4-β-D-glucopyranosamine monomers. It may also contain chitin (N-acetyl-1,4-β-D-glucopyranosamine) monomers in its composition. Due to the presence of N-acetyl groups in the composition, chitosan can have different degrees of deacetylation (DD). The amino group associated with acyl groups is less sensitive to protonation; as a result, the solubility of CS decreases. Since CS macromolecules contain a large number of amino groups, they can form a large number of hydrogen bonds. Therefore, it can bind many hydrophilic organic molecules, including SA. In addition, due to the protonation process, amino groups determine the cationic nature of CS NPs, which leads to a high affinity of the particles to a negatively charged biological membrane [64]. The large surface area of the NPs further increases their effectiveness. These factors lead to a significant ease in the penetration of NPs through the cell membrane [65,66,67,68,69,70,71]. Moreover, it is beneficial because the application of CS NPs provokes the innate defense response of the plants [72] against pathogen attack by producing antifungal hydrolases, phytoalexins, or by synthesizing lignin-like material [73]. At the same time, CS is completely biodegradable and does not damage the environment. The successful in vitro testing of a composite based on CS NPs and a furosemide-silver complex was reported [74]. The drug showed good antibacterial activity due to the ease of binding of Ag(I) ions to S-donor ligands present in bacterial proteins. CS provides drug transfer to the bacterial cell, and the furosemide molecule is an O-donor ligand for Ag^+^ ions, but is less preferred than a S-donor. The use of CS-SA/GM composite NPs to suppress the ototoxicity of gentamicin (GM) due to the ability of SA to bind free radicals was noted [75].

CS, itself can also have a positive impact on plant growth and defense mechanisms [76,77,78]. For example, the effect of SA, CS, lithovit^®^ (commercial name of a natural organic calcite carbonate and used as a foliar fertilizer), and potassium thiosulfate on the growth of tomato (*Solanum lycopersicum*) and protection against black rot (*Alternaria alternata*) pathogen were reported by El-Garhy et al. [16]. For in vitro tests, cultures of black rot pathogen were placed in solutions of the studied elicitors, and after seven days of incubation, the colony size was studied. Plants were sprayed with the elicitors at different growth stages. The concentration of lithovit^®^, potassium thiosulfate, and CS was 4.0 g/L, and SA was 1.5 g/L. SA was found to be able to inhibit the development of the black rot pathogen completely. CS, followed by SA was the most effective among the tested elicitors in controlling black rot in tomato. On the other hand, treatment with CS showed the highest SAR. This treatment resulted in a relatively strong expression of all seven stress-sensitive genes (Section 1) tested. At the same time, all elicitors showed high vegetative growth, fruit quality, and yield. Thus, the authors recommend combining use of CS and SA to protect tomatoes from black rot and increase yield.

**Figure 3:**
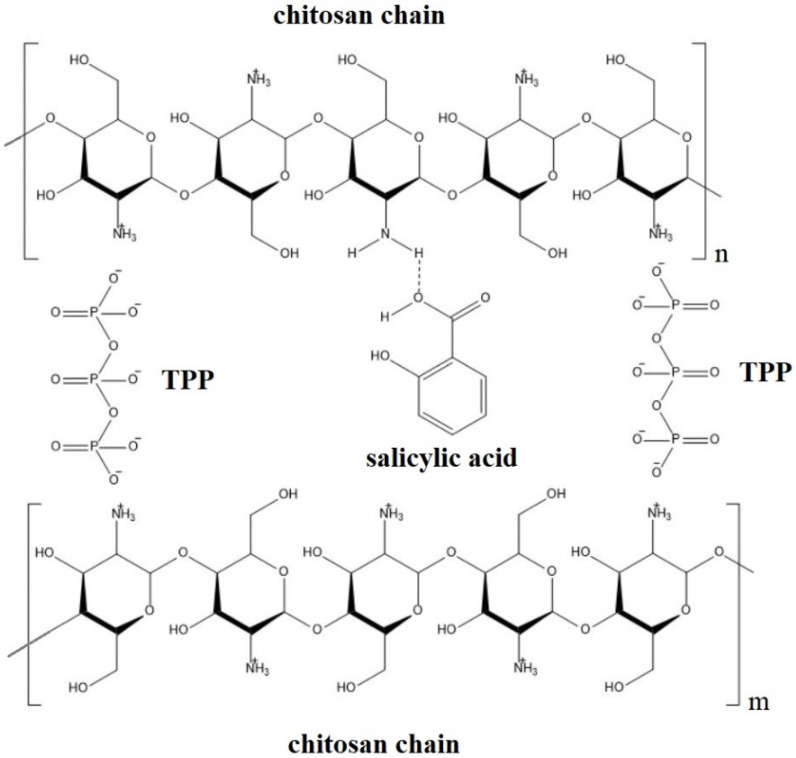
Hypothetical representation of the salicylic acid-chitosan complex structure. Adapted from [55].

Earlier studies [55,56,57] reported the development of a nanocomposite based on CS and SA. The ionic gelation method was used to prepare the nanocomposite. A CS solution in acetic acid was added to a mixture of SA and sodium tripolyphosphate. An acidic medium is necessary for the CS protonation of amino groups. Tripolyphosphate acts as a cross-linker of CS chains. As a result, the formation of a hydrogen bond between SA and CS chains contributes to the isolation of SA from the external environment by pyranose rings (Figure 3).

The slow hydrolysis of glycosidic bonds in neutral solutions causes the gradual release of phytohormone during plant growth and development [79]. Thus, even small amounts of the composite have a prolonged action. Positive effects of SA-loaded CS NPs (size 60–70 nm) obtained by ionic gelation technique were shown on the growth and protection of maize (*Zea mays*) [55] and wheat (*Triticum aestivum*) [57]. The obtained colloids had high stability and had a ζ-potential of +34.1 mV. ζ-potential is the electrical potential at the slipping plane, which separates the mobile fluid from the fluid that remains attached to the surface. The higher its absolute value, the more stable the colloid. The encapsulation efficiency (EE) was 80%, while the loading capacity of the NPs was estimated at 45 wt.%. The release of SA at pH 4.5 after 12 h and 96 h was 35.2% and 68.1%, respectively. The relatively fast release of SA in the first 12 h is attributed by the authors to the easy release of SA located on the NPs surface. After that, the authors recorded the relative slow release of SA, probably associated with the slow process of deeply located SA molecule diffusion.

Increased α-amylase and protease activity were found in maize seeds and wheat seedlings. This was confirmed by an increase in the content of starch and protein. Moreover, the activity of enzymes during treatment with the SA-CS NPs was higher than during treatment with pure CS or SA. It leads to an increase in seedling vigor index (SVI), and, consequently, to an increase in the shoot–root length and fresh weight (FW).

Another exhibition of the SA CS NPs influence was an increase in the activity of antioxidant and defense enzymes. After the second day of treatment, an increase in the activity of superoxide dismutase (SOD), catalase (CAT), peroxidase (POD), phenylalanine ammonia-lyase (PAL), and polyphenol oxidase (PPO) was observed. The apparent contradiction with the data on catalase and peroxidase inhibition can be explained by the presence of enzyme isoforms insensitive to SA [46] found, in particular, in maize [52]. The increase in enzyme levels is probably associated with the unusual protective function of SA under conditions of severe oxidative stress, which is able to neutralize excess H_2_O_2_ [36]. SOD and POD enzyme activity was more pronounced than CAT, PAL, and PPO enzymes. There was also a synergistic effect of the SA CS composite NPs compared to pure SA treatment. The high concentrations of NPs (above 0.11%, *w*/*v*) turned out to be less effective in neutralizing O_2_^−^ and H_2_O_2_ (most effective 0.04–0.08%, *w*/*v*). The marker of active lipid peroxidation under the action of ROS is malondialdehyde (MDA) [80]. Its high concentrations in tissues indicate oxidative stress, which leads to the destruction of the bilipid layer of cells and, as a result, to cell death. By increasing the activity of SOD, oxidative stress is reduced. The decrease in the concentration of MDA after the treatment of seedlings with NPs was noted, but high concentrations of SA CS NPs, on the contrary, lead to an increase in the MDA level. This fact correlates with a decrease in enzyme activity at composite concentrations above 0.11%, *w*/*v*. An increase in chlorophyll content was also found. This indicates a higher level of photosynthesis in the flag leaf and hence a greater sink strength [55].

Composite treatment was also found to result in proline accumulation in the flag leaf. Several studies [81,82,83,84] revealed a relationship between proline accumulation and plant resistance to stressors. It protects protein–lipid complexes by neutralizing hydroxyl radicals and ROS. Proline is involved in the complex gene regulation of various SOD isoforms, and one of the most common osmolytes that maintain the osmotic potential of the cell [85]. In addition, the formation of proline under stress conditions is a way to regulate the pH of the cytosol [86].

Effective inhibition of *Fusarium verticillioides* growth was also shown in in vitro experiments. It is an intracellular endophytic pathogen with symptoms during the flowering stage [87]. With foliar maize treatment, it was managed to control the post-flowering stalk rot (PFSR) development caused by *F. verticillioides*.

By the ionic gelation method, CS microparticles (MPs) loaded with salicylic acid was synthesized [56]. CS was obtained from the remains of shrimp [88] by extracting it from proteins, minerals, and pigments. The authors obtained chitosan with a M_w_ 1531 ± 372 kDa and DD above 87%. It was reported that chitosan with high M_w_ and DD has higher biocompatibility and slower release of active substances, while maintaining the transport function [89,90].

As a result, collapsed vesicular-shaped MPs (size about 1.6–3.4 μm) were formed. Microparticle shape caused the interaction of tripolyphosphate with protonated amino groups of chitosan. The cross-linker/polymer ratio plays a key role in particle shape formation [91,92]. The authors also studied the dependence of encapsulation efficiency on the SA concentration. It was noted that high concentrations of salicylic acid led to a decrease in the EE due to the competitive interaction of the salicylate ion with the tripolyphosphate ion when they bind to the protonated amino groups of chitosan [56]. SA concentrations of 1–5% (*w*/*w*) are optimal.

The kinetics of SA release were explored with the Equation (1) proposed by Korsmeyer and Peppas [93] to explain the mechanism for the SA release from the CS matrix:

(1) $$\frac{M^{t}}{M^{\infty }}=Kt^{n}$$

where *M^t^/M^∞^* is the fraction of the solute that was released at a certain time *t*; *K* is a constant linking the structural and geometric characteristics of the delivery system; coefficient *n* determines the mechanism of substance release for spherical particle shape. As reported by Costa and Lobo [94], *n* < 0.43 refers to Fick diffusion, and *n* between 0.43 and 0.85 refers to anomalous transport. If *n* > 0.85, this indicates the mechanism of controlled swelling of the polymer. Using the SA release curves, the authors estimated the value of *n* for all composites. This value corresponded to anomalous transport, i.e., a process that combines both the SA diffusion and the CS swelling.

The cytotoxicity of the obtained composites by estimating the effect of their various concentrations on the primary lettuce root (PR) length was also determined [56]. It turned out that high concentrations of composites (500 µg/mL) at any loading of SA have a toxic effect and significantly reduce the PR length. High loadings of SA (MP-20SA) also showed toxic effects at relatively low composite concentrations (50 µg/mL). Simultaneously, lower loadings of SA at the same concentration did not lead to a decrease in PR length but led to stimulation of root growth. This indicates a positive effect of SA on the growth of the root system. The researchers also revealed the toxicity of free CS compared to MPs. It may be caused by the high charge density of free CS due to the protonation of amino groups. These positive charges can act on the negatively charged bilipid layer of cell membranes, disrupting their functioning by changing their surface potential [95,96,97]. Tripolyphosphate in CS MPs partially neutralizes the charges of amino groups, resulting in a sharp decrease in cytotoxicity. On the other hand, low concentrations of composites at low SA loadings also demonstrated an increase in fresh root biomass. Thus, the positive effect of SA and CS MPs is dose dependent. High concentrations of both SA and the composite can lead to negative consequences, and it was also found with a significant increase in PR2 protein levels with low loadings of SA. The PR2 protein is associated with pathogenesis and is used as a marker of innate immunity [98]. The accumulation of protein indicates the activation of SA-dependent plant defense under biotic stresses.

### 2.2. Cellulose-Based Delivery Systems

**Figure 4:**
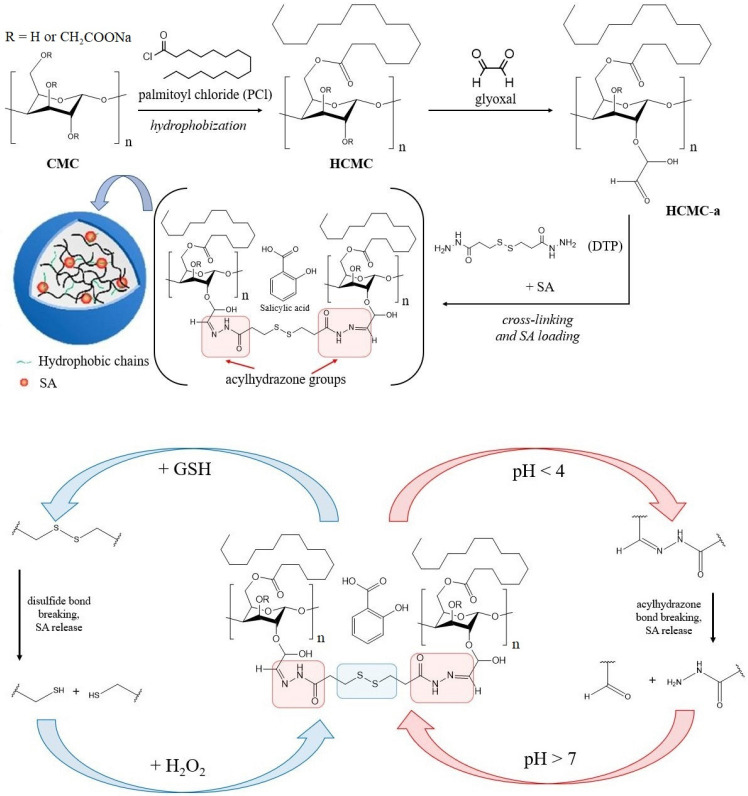
Cellulose-based nanogel synthesis process and its adaptive behavior in an environment with different pH and in the presence of glutathione. Adapted from [58].

The possibility of controlled pH- and redox-responsive release of agrochemicals from cellulose-based nanogel was explored [58]. The nanogel was obtained by a 3-step method (Figure 4).

In the first step, carboxymethyl cellulose (CMC) was esterified with palmitoyl chloride (PCl) to introduce hydrophobic chains into the cellulose (HCMC). Hydrophobicity is necessary for more efficient loading of agrochemicals that are poorly soluble in water. This fact was confirmed when evaluating the entrapment efficiency (EN%) and loading capacity (LC%) in comparison with a control sample without hydrophobic palmitic chains. The EN% and LC% of nanogels increased from 34.50% and 9.67% to 74.50% and 38.50%, respectively. After hydrophobization, the remaining etheric OR groups were condensed with glyoxal to introduce free aldehyde groups into cellulose (HCMC-a). The polysaccharide chains were then cross-linked with each other by forming acylhydrazone bonds with 3,3′-dithiobis(propionohydrazide) (DTP) in the manner of Schiff base. Simultaneously with cross-linking, in situ loading of SA was carried out. A composite was formed consisting of a cellulose carrier and a loaded phytohormone. Transmission electron microscopy (TEM) analysis showed the formation of spherical composite NPs 116 ± 42 nm in size.

However, NPs were able to aggregate. The degree of aggregation depended on the synthesis time: the longer the interaction proceeds, the stronger the aggregation. The maximum aggregate size recorded by the authors was 716 ± 50 nm after 20 min. The ultrasonic treatment made it possible to reduce the aggregate up to 443 ± 34 nm in size. A decrease in the size of aggregates promoted better uptake of NPs by plant roots and leaves, and also facilitated their transport in tissues [99]. The stability of the nanogel in sucrose solutions with a concentration of 0 mol/L–0.3 mol/L and 0.6 mol/L was also evaluated. In pure water, the swelling coefficient relative to the dried nanogel was 282%, and with an increase in the sucrose concentration up to 0.3 mol/L, the swelling coefficient decreased to 192% and did not change to 0.6 mol/L. Such high stability of the nanogel, even in relatively concentrated sucrose solutions, contributed to the preservation of its properties when used in soils with high salt content.

The choice of DTP as a cross-linker is due to two reasons. First, the resulting acylhydrazone bond –CH=N-NH-C(=O)– is unstable in an acidic environment. With a decrease in pH, they are able to hydrolyze with the destruction of the carrier structure, which leads to the release of SA. For example, it was shown that in neutral media, only 23% of SA is released slowly over 12 h. However, as the pH decreases, the rate increases (8 h), and the fraction of released SA increases, which is associated with the breaking of acylhydrazone bonds. With an increase in pH, broken bonds condense again to form the original structure. On the other hand, the cross-linker contains a redox-sensitive disulfide –S–S– group. In the presence of reducing agents, such as glutathione (GSH), it is capable of a break with the formation of thiol groups -SH. This process also leads to the destruction of the carrier matrix. Glutathione solution was used to mimic the in-vivo environment. It is known that GSH is one of the components of the antioxidant defense system in plants. It is the main factor determining their cellular redox status [100,101,102]. An increase in the level of glutathione occurs as a result of any external influences. A twofold increase in the concentration of glutathione leads to a twofold decrease in the rate of SA release. The addition of an oxidizing agent, such as hydrogen peroxide, to such a system makes it possible to re-form the disulfide bond. Thus, this cellulose nanogel is pH and redox sensitive. Analyzing the release curves of SA at both a pH 5.5 and GSH concentration of 0.1 mmol/L, the authors concluded that under these conditions, the diffusion–erosion kinetic model is realized. At the initial stage, Fick diffusion dominated in the surface layer, after which the combined effect of diffusion and erosion ensured a stable release of SA. The nanogel was also shown to be highly efficient in the sorption of heavy metals due to the coordinating properties of thiol and carboxyl groups.

### 2.3. Silica-Based Delivery Systems

**Figure 5:**
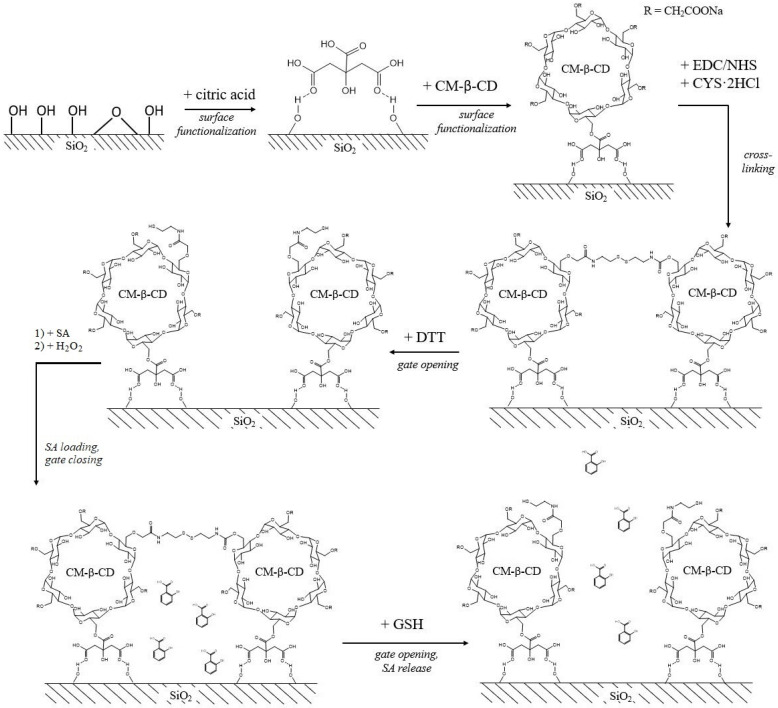
Scheme of the silica functionalization and SA loading/release processes. Adapted from [59].

Later, materials similar in functional properties based on silica NPs modified with thiol-carboxymethyl-β-cyclodextrin were synthesized with 20 nm particle size (Figure 5) [59]. The size is optimal for passing through the cell wall.

In the first stage, the silica NPs were coated with carboxymethyl-β-cyclodextrin (CM-β-CD). For stronger binding of the CM-β-CD to the silica surface, citric acid was used. Then, after carboxyl group pre-activation using N-hydroxysuccinimide (NHS) and 1-(3-(dimethylamino)propyl)-3-ethylcarbodiimide hydrochloride (EDC), the carboxymethyl groups were bound to cystamine (CYS·2HCl). Then, by introducing a reducing agent called dithiothreitol (DTT), they broke the disulfide bonds of cystamine with the formation of terminal thiol groups. SA was loaded into the pores of the silica matrix and the space of the network structure by free diffusion. After loading, the composite was treated with hydrogen peroxide, which again oxidized the thiol groups to disulfide ones. Thus, a redox-sensitive complex was formed, which, with a change in potential, for example, in the presence of GSH, could again destroy disulfide bonds with the SA release. After the release of SA, free thiol groups could act as chelates for binding heavy metal ions. The loading capacity of SA (214.24 mg/g) of the resulting composite turned out to be higher than pure nanosilica (7.32 mg/g) and CM-β-CD-modified SiO_2_ NPs without thiol (108.75 mg/g). An analysis of the kinetic curves of SA release showed that in pure water, n = 0.38, which corresponds to the Fickian diffusion mechanism. However, in a 5 mM GSH solution, n = 0.88, which corresponds to a complex mechanism including swelling and erosion processes.

**Figure 6:**
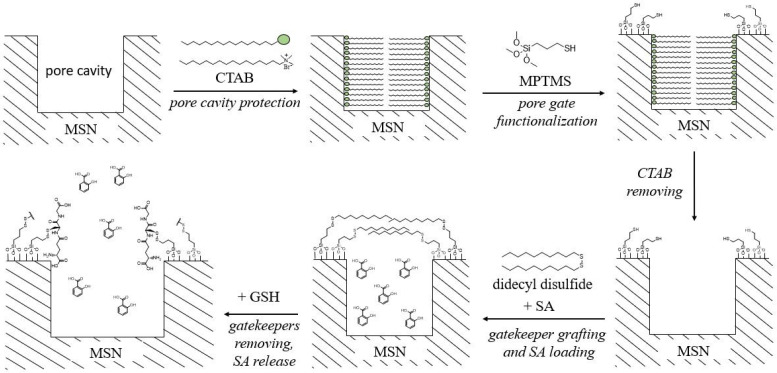
Synthesis scheme and structure of NPs functionalized with decanethiol MSN loaded with SA. The process of the removal of the gatekeepers under the influence of GSH in an aqueous solution. Adapted from [60].

In an earlier study [60], mesoporous silica nanoparticles (MSN) were modified with decanethiol, and the mesopores were used to load salicylic acid. Figure 6 shows the synthesis scheme.

At the first stage, thiol groups were grafted onto mesoporous silica containing the CTAB (cetyltrimethylammonium bromide) matrix using 3-mercaptopropyltrimethoxysilane (MPTMS). CTAB was necessary for functionalization with thiols to occur only at the entrance to the mesopores to preserve their size. Then CTAB was removed to free the inner surface of the mesopores. After that, decanethiol fragments were grafted onto free SH-groups using didecyl disulfide to form disulfide bonds. Simultaneously, the mesopores were loaded with salicylic acid. Decane chains acted as gatekeepers of mesopores, preventing SA molecules from entering cavities. The addition of GSH to the system led to the removal of decane chains and the release of salicylic acid molecules from the pore cavity. The n values of Equation (1) in 5 mM and 10 mM GSH solutions were 0.32 and 0.39, respectively, indicating a Fickian diffusion mechanism after gate opening.

The effect of the SA slow release was studied by the authors on *Arabidopsis thaliana* through the expression of PR-1 along with the level of GSH accumulation. Seeds, after sterilization, were placed in plates containing the studied composites. Plants without treatment and plants treated with free salicylic acid and MSN were used as control experiments. Only when the plant was treated with a composite was the expression of the PR-1 gene detected on all days of observation. At the same time, the slow release of salicylic acid provoked an increase in the GSH level, which in turn increased the rate of removal of the gatekeepers. This cycle provided a monotonic stable increase in GSH levels, in contrast to the use of a free matrix and free salicylic acid. Thus, the composite provides prolonged protection of the plant from biotic stress. A similar study with the same findings was conducted on *Ananas comosus* [61].

### 2.4. CeO_2_-Based Delivery Systems

The effect of cerium dioxide NPs on plants was studied in detail in the literature. It plays an important role in enhancing the physiological responses of plants, such as gene expression [103,104], photosynthesis efficiency [105], oxidative potential [106,107], water absorption [108], and nutrient absorption [104]. The authors of [62] used cerium dioxide CeO_2_ as a carrier for the SA delivery. They studied the effect of the combined application of cow manure and foliar treatment with CeO_2_: SA-NPs on the growth and physiological responses of *Aloe vera*. Cerium dioxide was obtained by ultrasonic treatment of a solution containing cerium (III) nitrate and urea, followed by calcination at 850 °C of the obtained precipitate. Loading with SA was carried out by simple mixing of NPs and SA solution in various concentrations. The size of the obtained CeO_2_: SA-NPs (30 nm–80 nm) was estimated by TEM and DLS methods.

The study found that 10% and 20% manure content in the soil and foliar application to *Aloe vera* showed the most positive results. In particular, a significant increase in the fresh/dry weight of the plant, diameter, length, and number of leaves, as well as the chlorophyll content and their gel fresh/dry weight, elemental composition was noted [62]. In *Aloe vera* leaf gel, the authors also found an increase in the content of aloin, phenols, and flavonoids. The harvest index increased, as did, consequently, the economic yield. The synergy of manure and composite effects was confirmed by control experiments using distilled water, free CeO_2_ NPs, and free SA.

### 2.5. SA-Based Delivery Systems

SA itself in the NPs form can be used as a delivery matrix [63]. The effect of SA NPs on *Catharanthus roseus* under drought stress was studied. SA NPs were obtained by sonication of a commercial SA in absolute ethanol. The particle size was 5.17 nm–17.30 nm.

In this study, SA NPs and bulk SA were introduced into plants by foliar application. The use of SA under drought stress conditions led to an increase in plant growth, in particular, an increase in leaf area index, plant length, shoot and root dry weight, and an increase in the shoot/root ratio was noted. An increase in the content of water, chlorophyll, carotenoids, and alkaloids was also noted. The scientists also observed an increase in the expression levels of drought tolerance-associated genes: WRKY1, WRKY2, WRKY40, MPK1, MPK6, LEA, and MYC2. The use of SA nanoparticles turned out to be preferable. The functional effect of the SA nanoform is a higher dissolution rate due to the larger active surface area. As the study showed, for the effective use of SA in the bulk particles form, it is necessary to use high concentrations, while NPs have a significant effect on plants even at very low concentrations.

## 3. Conclusions

In summary, it should be noted that in the last 5 years, new promising methods for the delivery and controlled release of salicylic acid were increasingly developed. As can be seen from Section 2.1, chitosan-based delivery systems are pH sensitive and are able to gradually hydrolyze in slightly acidic or slightly alkaline environments, providing a slow controlled release of salicylic acid. Moreover, the larger the size of carrier nanoparticles, the more prolonged effect the composite has on the plant. In addition, the chitosan matrix is biodegradable and completely non-toxic, which has a positive effect on the purity of food products derived from plants. The composite using leads to an increasing in the fresh weight of the plant, an increasing in protection, and to the death of the black rot pathogen. A composite based on cellulose nanogel acts more complex (Section 2.2). It is not only pH-sensitive, but also redox-sensitive due to the presence of disulfide and acylhydrazone bonds. It is known that glutathione determines the redox status of cells; therefore, in its presence, disulfide bonds are broken with the release of salicylic acid. The free thiol groups can then act as chelators to tightly bind heavy metal ions. The idea of redox-sensitive matrices was extrapolated to inorganic carrier nanoparticles (Section 2.3). Thus, composites based on silica gel nanoparticles modified with thiols also provided a slow release of salicylic acid. After release, increased expression of the PR-1 gene was observed. Section 2.4 shows that uncoated inorganic matrices can also serve as effective carriers for the delivery of phytohormones. Thus, composites based on cerium dioxide, when applied, caused a whole range of positive reactions in plants, including an increase in fresh weight and root/leaf length, accumulation of chlorophyll, flavonoids, aloin, and phenols as natural antioxidants. Even salicylic acid itself in the form of nanoparticles can act as a delivery agent (Section 2.5). Due to its lower solubility, it is capable of the slow release of salicylic acid. The effects of the use of nanoparticles also turned out to be diverse, from increasing plant growth to increasing the content of alkaloids, carotenoids, chlorophyll, and water. The effect of all considered composites was compared to the effect of free salicylic acid and a free carrier. In all cases, the composite remained the most effective, proving the synergy of the action of its components. Despite advances in the development of salicylic acid delivery systems based on nanoparticles, not only further research and development of new approaches in this area are still needed, but also the widespread introduction of ready-made solutions directly in agriculture, together with the collection and analysis of the resulting statistical data. This will help to reveal possible shortcomings of existing delivery systems and make appropriate changes in the composition and structure of composites.

## Figures and Tables

**Table 1:** Summarizing of reported SA delivery systems.

Carrier	Size, nm/Shape/	Plant	Effect	Ref.
Chitosan	~368.7 by dynamic light scattering (DLS) 60–70 by transmission electron microscopy (TEM) /spherical/	Maize [*Zea mays*]	inhibits the: − mycelium growth (*Fusarium verticillioides*); − post flowering stalk rot (PFSR). increase in the: − plant growth; − antioxidant and defense enzyme activity; − ROS destruction rate.	[55]
	1570–3350 (TEM) /collapsed vesicular/	Butterhead lettuce [*Lactuca sativa* cv. Reina de Mayo]	inhibits the: − primary root (PR) length at high SA concentration. increase in the: − fresh root biomass at low SA concentration; − plant defense.	[56]
	~368.7 (DLS) 60–70 (TEM) /spherical/	Wheat [*Triticum aestivum*]	increase in the: − shoot-root length; − fresh weight (FW); − seedling vigor index (SVI); − antioxidant and defense enzyme activity; − ROS destruction rate.	[57]
Cellulose nanogels	~116 (TEM) /spherical/	-	− pH- and redox-sensitivity of nanogel; − high stability in sucrose solutions; − possibility of sorption of heavy metals.	[58]
Thiol-CM-β-CD-modified SiO_2_ NPs	~20 (TEM) /spherical/	-	− pH- and redox-sensitivity of composite; − possibility of sorption of heavy metals.	[59]
Decanethiol-modified SiO_2_ NPs	~20 (TEM) /spherical/	*Arabidopsis* *thaliana*	− redox-sensitivity of composite; − increase in PR-1 gene expression.	[60]
Decanethiol-modified SiO_2_ NPs	20–30 (TEM) /spherical/	*Ananas comosus*	− redox-sensitivity of composite; − increase in PR-1 and PR-5 genes expression.	[61]
CeO_2_	30–80 (TEM, DLS) /octahedral/	*Aloe vera*	increase in the: − plant fresh/dry weight; − leaves diameter, length and number; − chlorophyll content; − gel fresh/dry weight; − elemental composition; − content of aloin, phenols and flavonoids; − harvest index.	[62]
SA NPs	5.17–17.3 (TEM) /spherical/	*Catharanthus roseus*	increase in the: − plant growth; − shoot/root dry weight; − shoot/root ratio; − leaf area index; − chlorophyll, water, carotenoids and alkaloids content; − drought tolerance-associated genes level.	[63]

## Data Availability

Enquiries about data availability should be directed to the authors.

## Abbreviations

ACS	catalytic hydrolase-2;
CAT	catalase;
CMC	carboxymethyl cellulose;
CM-β-CD	carboxymethyl-β-cyclodextrin;
CS	chitosan;
CTAB	cetyltrimethylammonium bromide;
CYS	cystamine;
DD	degree of deacetylation;
DLS	dynamic light scattering;
DTP	3,3′-dithiobis(propionohydrazide);
DTT	dithiothreitol;
EDC	1-(3-(dimethylamino)propyl)-3-ethylcarbodiimide hydrochloride;
EE	encapsulation efficiency;
EN	entrapment efficiency;
FW	fresh weight;
GM	gentamicin;
GSH	glutathione;
HCMC	hydrophobic carboxymethyl cellulose;
HCMC-a	hydrophobic carboxymethyl cellulose with aldehyde groups;
LC	loading capacity;
LEA	late embryogenesis-abundant protein;
LOXD	lipoxygenase D;
MAPK	mitogen-activated protein kinase;
MDA	malondialdehyde;
MPs	microparticles;
MPTMS	3-mercaptopropyltrimethoxysilane;
MSN	mesoporous silica nanoparticles;
NHS	N-hydroxysuccinimide;
NPs	nanoparticles;
PAL	phenylalanine ammonia-lyase;
PCl	palmitoyl chloride;
PFSR	post flowering stalk rot;
POD	peroxidase;
PPO	polyphenol oxidase;
PR	primary root;
PR-1	pathogenesis-related protein 1;
RAP	ethylene-responsive transcription factor 3;
PINII	proteinase inhibitor II;
ROS	reactive oxygen species;
SA	salicylic acid;
SAR	systemic acquired resistance;
SOD	superoxide dismutase;
SVI	seedling vigor index;
TEM	transmission electron microscopy;
XET-2	xyloglucan endotransglucosylase 2.
